# An Objective-Based Entropy Approach for Interpretable Decision Tree Models in Support of Human Resource Management: The Case of Absenteeism at Work

**DOI:** 10.3390/e22080821

**Published:** 2020-07-27

**Authors:** Gonen Singer, Izack Cohen

**Affiliations:** Faculty of Engineering, Bar-Ilan University, Ramat-Gan 52900, Israel; izack.cohen@biu.ac.il

**Keywords:** human resource management, absenteeism, ordinal classification, information gain, decision tree, interpretable machine learning models

## Abstract

The negative impact of absenteeism on organizations’ productivity and profitability is well established. To decrease absenteeism, it is imperative to understand its underlying causes and to identify susceptible employee subgroups. Most research studies apply hypotheses testing and regression models to identify features that are correlated with absenteeism—typically, these models are limited to finding simple correlations. We illustrate the use of interpretable classification algorithms for uncovering subgroups of employees with common characteristics and a similar level of absenteeism. This process may assist human resource managers in understanding the underlying reasons for absenteeism, which, in turn, could stimulate measures to decrease it. Our proposed methodology makes use of an objective-based information gain measure in conjunction with an ordinal CART model. Our results indicate that the ordinal CART model outperforms conventional classifiers and, more importantly, identifies patterns in the data that have not been revealed by other models. We demonstrate the importance of interpretability for human resource management through three examples. The main contributions of this research are (1) the development of an information-based ordinal classifier for a published absenteeism dataset and (2) the illustration of an interpretable approach that could be of considerable value in supporting human resource management decision-making.

## 1. Introduction

Absenteeism, in contrast to planned time off, may cause significant disruptions to organizations and may affect their productivity and profitability. Absenteeism and its effects may be controlled by equipping human resource management with the ability to predict which groups of employees are most prone to absenteeism. The current research focuses on providing such predictions via interpretable, information-based, machine learning models. The suggested approach may serve human resource management in conducting analyses, developing policies, and, eventually, in combatting absenteeism and its effects.

Large-scale research on absenteeism can be traced back to the highly cited paper of Porter and Steers in 1973 [1]. They group the factors that affect absenteeism into (1) organizational, (2) immediate work-related, (3) job-related, and (4) personal. The numerous studies that followed have generally explored this set of factors. For example, Soriano et al. [2], who analyzed data from 1346 indoor office employees, confirmed that sets of factors that include “job satisfaction and health” and “job satisfaction and affective well-being” are significantly correlated with absenteeism. Other studies observed correlations between absenteeism and workload [3], age [4,5,6], work performance [7], and body characteristics [8,9]. Accordingly, in this research we use a dataset that includes factors that have been previously reported as significantly associated with absenteeism.

As argued by Tewari et al. [10], machine learning approaches, which are becoming an increasingly popular research tool, are highly suited to the analysis of absenteeism data. They are more appropriate than conventional statistical approaches, such as hypotheses testing and ordinary least square regression, due to the fact that the rate of absenteeism is a skewed, truncated measure, and thus it does not follow a normal distribution [10]. Furthermore, the conventional statistical approaches focus on identifying features that are correlated with absenteeism across the whole dataset in contrast to the suggested entropy-based approach that discovers absenteeism patterns, as we demonstrate in this paper.

We now briefly review the main findings of machine learning models that have analyzed absenteeism, and in so doing, we highlight the contributions of the present study. To enable traceability and facilitate comparison with previous research, we use a dataset that was first introduced by [11] and has been subject to fairly extensive research (see, e.g., in [12,13,14,15]). Wahid et al. [12], for example, employed various models, such as Decision Tree, Tree Ensemble, Gradient Boosted Tree, and Random Forest, to predict the absence time. For the purpose of classification analysis, they transferred the absence time, which is recorded in hours, into four classes: “hours”, “days”, “weeks”, and “not absent”. In terms of accuracy, their classification models achieved values of 79–82%. In [13], the rate of absence was discretized into just two classes: less than or equal to 5 hours, or greater than 5 hours. Dogruyol and Sekeroglu [14] and Araujo et al. [15], on the other hand, treated the target variable—absenteeism in hours—as a continuous variable. They analyzed the same dataset using models such as the Backpropagation (BP) Neural Network, the Radial-Basis Function Neural Network (RBFNN), and the Long Short-Term Memory Network (LSTM). While the BP method did not perform well, RBFNN and LSTM achieved R^2^ values of 0.90 and 0.99, respectively. Note, however, that these analyses used the “reason for absence” as a feature, which although highly correlated with absence (i.e., every instance with an empty field for “reason for absence” obtains a value of 0 for the feature “absenteeism time in hours”), is not known before the absenteeism event. The main contributions of this research include the introduction of a new information measure, known as objective-based entropy, which considers the ordinal nature of the target (in this case, absenteeism). In addition, we highlight the value of interpretable models as decision support tools for human resource management. The combination of interpretable modeling and a metric that considers ordinal data makes our model valuable for analyzing and predicting absenteeism patterns.

## 2. Materials and Methods 

We begin by describing the environment and the dataset. We then present the theoretical features of the proposed objective-based entropy measure and describe its implementation in interpretable decision tree models for selecting the most useful attributes for explaining absenteeism at work.

### 2.1. The Dataset and Data Preparation 

We use a workplace absenteeism dataset for the period between July 2007 and July 2010 from a courier company in Brazil. This 740-sample dataset, which is available at the UCI Machine Learning Repository [11], has been subject to previous investigations using various machine learning models (see, e.g., in [12,13,14,15]). Table 1 lists the 21 features of the dataset that reflect work-related and personal factors. As discussed in Section 1, we omit from our analysis the feature “reason for absence”, which is highly correlated with absenteeism but only known in retrospect. In addition, we omit the feature “id”, as it plays no significant role in the prediction of specific absenteeism events. 

Following the International Labor Standards on Working Time by the International Labor Organization (ILO), we apply the standard of working 8 hours in a day or 40 hours in a week to categorize the target feature “absenteeism time in hours” into four categories: “not absent”, “hours”’, “days”, and “weeks”; see Table 2 for the classes and their respective probabilities within the dataset. 

For model evaluation, the data are split into a training dataset (80% of the data, which corresponds to 592 samples) and a testing dataset (20%, which comprises the remaining 148 samples). In a class-imbalanced dataset, a random split can result in different class distributions between the training and testing datasets, even in a testing set that does not include instances of a minority class. To minimize variation between the two datasets, the samples are selected such that the distribution of class probabilities is similar in both datasets [16]. To prevent biased learning due to imbalanced class distributions [17], we apply a Synthetic Minority Oversampling Technique (SMOTE) algorithm [18] to the training dataset, which results in a dataset of 1360 instances with a 25% probability per class. Note that, when applying an oversampling technique to a class-imbalanced dataset, the k-fold cross-validation technique can be computationally expensive and difficult to implement; thus, techniques such as the holdout method, which we implement in this study, are typically used instead [19]. Table 3 presents the class distribution of the training dataset before and after SMOTE implementation. A further preprocessing step, which is needed to prepare the data for our new entropy measure (to be explained in the next section), consists of discretization of the continuous dataset features. Finally, we can formulate the training dataset as $D=\{(x_{t},y_{t}),t=1,2,\ldots ,T\},$ where $x_{t}=\left[v_{t,1},v_{t,2},\ldots ,v_{t,K}\right]$ denotes a sample, *t*, in the dataset, defined by a vector of values, $v_{t,k}$, for each of its k=1,…,K features (e.g., if the age feature k for sample t is 36, then $v_{t,k}=36$). Let us denote the number of distinct values for each feature k as $N_{k}$, with the values themselves denoted by $a_{k,i},\;\forall i=1,\ldots ,N_{k}$; then, $v_{t,k}\in \{a_{k,i}:\;i=1,\ldots ,N_{k}\},\;\forall k.$ As shown in Table 2, the value of absenteeism in hours,y, is classified into one of four possible classes denoted by the random variable $c\in \left\{c_{1},c_{2},c_{3},c_{4}\right\}$, where $c_{1}$ is the class that does not exhibit absenteeism and $c_{4}$ is the class with the highest level of absenteeism (“weeks”). The respective probability of being in class $c_{i}$ is defined by $P(c_{i})$. We define the value of a class $V\left(c_{i}\right)$ as an increasing function with the value of absenteeism, such that $V\left(c_{i}\right)<V\left(c_{j}\right)\;\forall i<j$ and $V\left(c_{i}\right)=i$.

Following data preparation and preprocessing, the training dataset includes T=1360 samples, K=18 features, and one target feature which belongs to one of the four absenteeism classes. We use this dataset to compare ordinal and non-ordinal classifiers as presented in Section 3. 

### 2.2. Objective-Based Entropy 

Let us formulate Shannon’s entropy [20] as follows,

$$H(c)=-\sum_{i=1}^{n}P(c_{i})\log_{b}P(c_{i}),$$

where $P(c_{i})$ is the probability of being in class $c_{i}$ and b=2 in the present paper (i.e., bits). For readability, we omit the subscript b from future equations.

Obviously, the entropy value, which is determined exclusively by the probability values, is insensitive to the allocation of these probabilities to the classes. Consider, for example, two different probability distribution functions, (0.6, 0.3, 0, 0.1) and (0.6, 0.1, 0, 0.3), for the respective absenteeism classes (“not absent”, “hours”, “days”, and “weeks”)—the entropy value for these two scenarios is the same, H(c)=1.3. Nevertheless, a human resource manager would judge these as two significantly different scenarios; in the former, 90% of the instances are either not absent or are absent for less than a day, while in the latter scenario, 30% of the employees are absent for more than a week. The current research develops an objective-based entropy measure that distinguishes between such scenarios. It generalizes the concept of the weighted entropy measure in [21] and allocates a weight to each category based on the difference in class value with respect to a selected class $c^{s}$, where *s* represents the statistic that defines the selected class. If, for example, we select the class with the maximum value, then $c^{\max}=\underset{c_{i}}{\arg\max}V(c_{i})$, whereas if we select the most probable class, then $c^{\mathrm{mode}}=\underset{c_{i}}{\arg\max}P(c_{i})$.

We define the objective-based entropy (*OBE*) measure over dataset D, and for selected class $c^{s}$, as

(1) $$OBE(c^{s},D)=-\sum_{i=1}^{n}w(c_{i})P(c_{i})\log P(c_{i}),\;0\leq w(c_{i})\leq 1,$$

where

(2) $$w(c_{i})=\frac{{\left|V(c_{i})-V(c^{s})\right|}^{\alpha }}{\sum_{j=1}^{n}{\left|V(c_{j})-V(c^{s})\right|}^{\alpha }},\;\forall i.$$

Thus, $\sum_{i=1}^{n}w(c_{i})=1$ and α>0 is a normalization factor that biases the weights’ distribution over the different classes. For example, as α increases, the weights of the classes with values that are distant from the value of the selected class $c^{s}$ get larger. Note that as $\alpha \to 0^{+},\;w(c_{i})\to \frac{1}{n}\;\forall i$; therefore, each class is assigned the same weight and the *OBE* is similar to Shannon’s entropy, up to a factor. On the other hand, for large values of the normalization factor, say α→∞, only the class with the largest difference $\left|V(c_{i})-V(c^{s})\right|$ counts, as its weight $w(c_{i})\to 1$, while the weights of the other classes tend to zero. Thus, the normalization factor is a hyperparameter that can be tuned (e.g., via a grid search) for a given predictive modeling problem; this is the approach that we use in Section 3.

In other words, the *OBE* measure distinguishes between probability distributions with similar probability values but different assignments to classes. For example, assume that we wish to calculate *OBE* with respect to $c^{\max}$. Then, for a given value of α (e.g., 2), distributions with higher probability values for the class without absenteeism $c_{1}$ (e.g., $P(c_{1})>P(c_{3})$) would yield higher *OBE* values than scenarios in which the class probabilities are interchanged (e.g., $P(c_{3})\leftarrow P(c_{1}),\;P(c_{1})\leftarrow P(c_{3})$). Thus, by selecting class $c^{s}$, one can tune the *OBE* to identify a variety of probability distributions. By way of illustration, Table 4 presents the values of the standard Shannon’s entropy and the objective-based entropy with α=2 for selected classes $c^{\max}$ and $c^{\mathrm{mode}}$. In contrast to Shannon’s entropy, the *OBE* distinguishes between the two probability distributions. Note that when the probability distribution is skewed towards the selected class, the *OBE* value is lower. This intuitive explanation can guide the selection of $c^{s}$. For example, if one prioritizes the accurate classification of classes with high absenteeism values, then the class $c^{\max}$ is preferred to the class $c^{\min}$.

The next section demonstrates how to use the *OBE* to identify the features with the highest information gain for decision tree models. 

### 2.3. Objective-Based Information Gain (OBIG) for Selecting the Features with the Greatest Explanatory Value in a Decision Tree Model

In this section, we develop the objective-based information gain (hereafter, *OBIG*) measure for selecting the branching attributes in any decision tree model. Let us formulate the *OBIG* from the partitioning of dataset D over a feature k that has $N_{k}$ unique values as

(3) $$OBIG_{k}(c^{s},D)=OBE(c^{s},D)-\sum_{r=1}^{N_{k}}\frac{\left|D_{r}\right|}{\left|D\right|}\cdot OBE(c^{s},D_{r}),$$

where $OBE(c^{s},D)$ is defined in Equation (1), and the second expression on the right-hand-side (RHS) of Equation (2) is the *OBE* that follows from the partition over feature k. $\frac{\left|D_{r}\right|}{\left|D\right|}$ represents the frequency of the *r*th distinct value within the dataset for feature k and its respective $OBE(c^{s},D_{r})$ value. Similarly to the conventional information gain measure, the objective-based information gain is overly sensitive to the number of values of attribute k,$N_{k}$. Thus, in the case where there are large variations in $N_{k}$ among the features, we normalize the information gain in Equation (2) by dividing its value by the information generated from splitting the dataset into $N_{k}$ partitions (for an illustration of this approach in a C4.5 decision tree, see in [21]). This calculation results in the objective-based information gain ratio (OBIGR):

(4) $$OBIGR_{k}(c^{s},D)=\frac{OBIG_{k}(c^{s},D)}{H_{k}(D)}.$$

As our focus is on the CART model, which has shown good results in our preliminary experiments and branches via binary splitting at each node, we do not use Equation (3) in the present study. In other words, for the CART, $N_{k}=2$ at each node and the feature with the highest *OBIG* is selected via Equation (2).

### 2.4. Interpretable Classification Models in the Context of Absenteeism

Our focus on interpretable models is motivated by the superior trust that human beings have in such models, meaning that they tend to be preferred over non-interpretable models [22,23,24]. In fact, it has been argued that interpretable models should be favored over non-interpretable models with comparable or even slightly better performance [25,26]. In the context of this paper, where the goal is to devise an effective intervention program for reducing absenteeism at work, these strategies must be based on an understanding of absenteeism patterns and their respective employee profiles. Most previous studies about absenteeism at work employed non-interpretable models, such as Neural Networks, Random Forest, and Support Vector Machines. An exception is the study of Wahid et al. [12], which implemented two types of interpretable decision trees. We note, in passing, that our study also departs from previous research by omitting the feature “reason for absence”, as this feature is not known in advance of the absenteeism event and thus cannot be used to predict it. Moreover, most organizations do not record the medical reason for absenteeism, due to privacy and ethical considerations. The *OBIG* decision trees that we develop give rise to a set of rules that may shed light on the conditions and possible reasons for absenteeism, without requiring knowledge of machine learning models on the part of the user (i.e., human resource personnel). 

## 3. Results

### 3.1. A Comparison Between Interpretable Ordinal and Non-Ordinal Classifiers

This subsection compares the performance of the proposed *OBIG*-based ordinal CART model with popular non-ordinal alternatives, some of which have been previously applied to the absenteeism at work dataset (see, for example, in [12]). The ordinal algorithms were developed using the programming language Python, and the non-ordinal algorithms were implemented with the Scikit-learn library in Python. In light of our goal to identify absenteeism patterns, i.e., conditions and possible reasons for absenteeism, we apply the OBE-based CART models with selected classes $c^{\max}$ and $c^{\mathrm{mode}}$ rather than $c^{\min}$, as the latter represents the “not absent” class and thus leads to inferior classification results.

For benchmarking purposes, we calculate five measures of performance of the classification models: F-score, Precision, Recall, Accuracy, and Area Under the Curve (AUC) [27]. We also compute the Mean Square Error (MSE) and Kendall’s Correlation Coefficient, $\tau_{b}$, which are common performance measures for ordinal classification models [16,28]. These performance measures are presented in Table 5 for eight models (two ordinal and six non-ordinal), with the best performance values highlighted in bold. It can be seen that the ordinal CART model that is based on $OBE(c^{\max})$ yields the best performance of all the models for six out of seven indices. Additionally, the two ordinal CART models yield better performance than their non-ordinal counterpart, namely, the conventional CART. 

**Figure 1:**
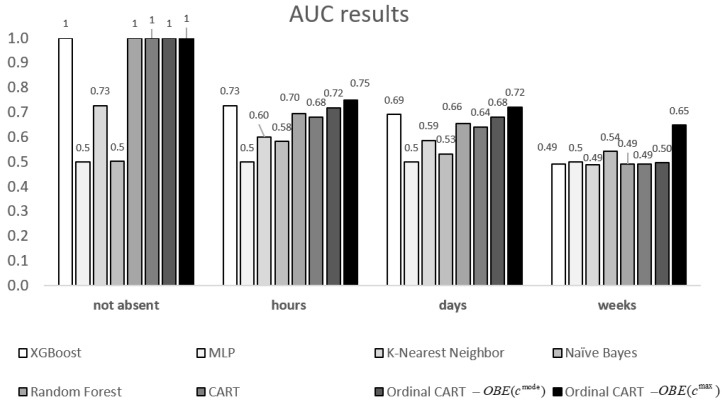
A comparative graph of Area Under the Curve (AUC) values (*y*-axis) for different learning models as a function of absenteeism classes (*x*-axis).

Figure 1 illustrates the AUC values obtained for each absenteeism class for each model. Let us focus on the highest absenteeism class (“weeks”), as it is important to identify the characteristics of those who are most susceptible. It can be seen that the ordinal CART $OBE(c^{\max})$ yields significantly better results for this class than the other models, with an AUC value that is larger than that of its closest competitor—the Naïve Bayes model—by 20% (AUC = 0.65 and 0.54, respectively). The ordinal CART $OBE(c^{\max})$ model also achieves the highest AUC values for the “hours” and “days” classes. 

To recapitulate, the overall performance of the proposed objective-based ordinal CART, based on the maximum desired output, outperforms the other models. It yields a decision tree with 123 leaves, 245 nodes, and a depth of 17. In the next section, we highlight the interpretability of the ordinal CART model, which is perhaps its most significant advantage, as it would enable human resource managers to extract patterns and insights that can be transformed into actionable policies. 

### 3.2. The Practical Value of the Interpretable Ordinal CART—Examples of Identified Patterns 

We illustrate the importance of interpretability by presenting three specific examples of patterns out of the many that have been revealed in the dataset. These patterns have been discovered by the ordinal CART algorithm, but not by its conventional counterpart that uses the classical entropy measure. These so-called patterns can be thought of as subgroups of employees that share common characteristics (in terms of the features in Table 1) and that result in the same class of absenteeism. In contrast to black-box models, the human resource manager can examine these patterns, which will allow them to discover both intuitive and counterintuitive phenomena and make data-informed decisions. From a practical perspective, the identified patterns can be used to devise intervention programs for reducing absenteeism at work, based on those common, subgroup characteristics. It is also worth noting that the ordinal CART model would enable a similar interpretation process to be applied to other challenges in both human resources and other domains.

**Example** **1.** **The relationship between age and the level of absenteeism.**

**Figure 2:**
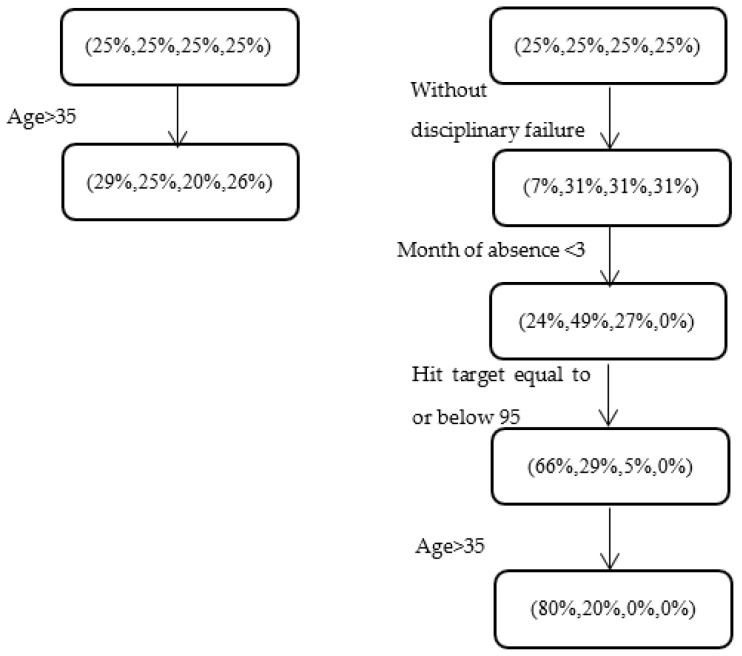
Relationship between age and absenteeism at work for different subgroups of employees. The LHS and RHS respectively show (i) a “simple” partition by age and (ii) a series of patterns revealed by our ordinal CART model.

This example was chosen as absenteeism has been reported to decrease with age (see, e.g., in [4]). However, as seen from the left-hand side (LHS) of Figure 2, a “simple” partition by age does not reveal any clear pattern of absenteeism. The right-hand side (RHS), on the other hand, which is produced by our model, presents a more refined picture. We observe that when the period of absence is at the beginning of the year, employees without a disciplinary failure indication, and with a relatively poor performance record, are distributed among the absenteeism classes according to (80%, 20%, 0%, 0%), if they are above 35 years old. This interesting pattern, which corresponds to a subgroup of 45 dataset instances, is significantly different (with *p*-value <0.001) from the distribution obtained for the full set of instances in which the employees are above the age of 35 years (29%, 25%, 20%, 26%). 

We emphasize that the interpretation of the results of our model should be carried out on a case-by-case basis, using the organization’s human resource management experts. Nevertheless, we offer a possible explanation for this general finding: It could be the case that employees above the age of 35 with performance target rates less than or equal to 95 (note that 58% of the instances in the training dataset had a target rate less than or equal to 95) worry more about their jobs during the initial months of the year than the average employee in this age group; therefore, they exhibit less absenteeism. This pattern, which was easily uncovered by our model, may be also deduced by integrating the following results from previous studies, although this process would be considerably more demanding and time-consuming for human resource managers. (1) A negative correlation between age and absence frequency [4,5,6]; (2) employees who have larger families and greater financial responsibilities are less likely to stay at home as a result of a minor illness [29,30]; and (3) following the logic in [7], employees with lower performance target rates may feel insecure and are thus less likely to be absent from work. When examining a pattern that is the same as that shown on the right-hand side except for a difference at the final level (i.e., the subgroup only includes employees who are younger than 35), the revealed class distribution is (9%, 64%, 27%, 0%), which is significantly different from the (80%, 20%, 0%, 0%) distribution obtained for employees above the age of 35 years. Thus, a possible intervention program would begin by administering a survey to the employees about their commitment to the organization and their attitudes towards improving their performance. The aims of the survey would be to understand the reasons that cause both subgroups to differ in terms of their propensity for absenteeism, and ultimately, to develop a prevention strategy for the younger subgroup.

**Example** **2.** **The relationship between body characteristics and the level of absenteeism.**

**Figure 3:**
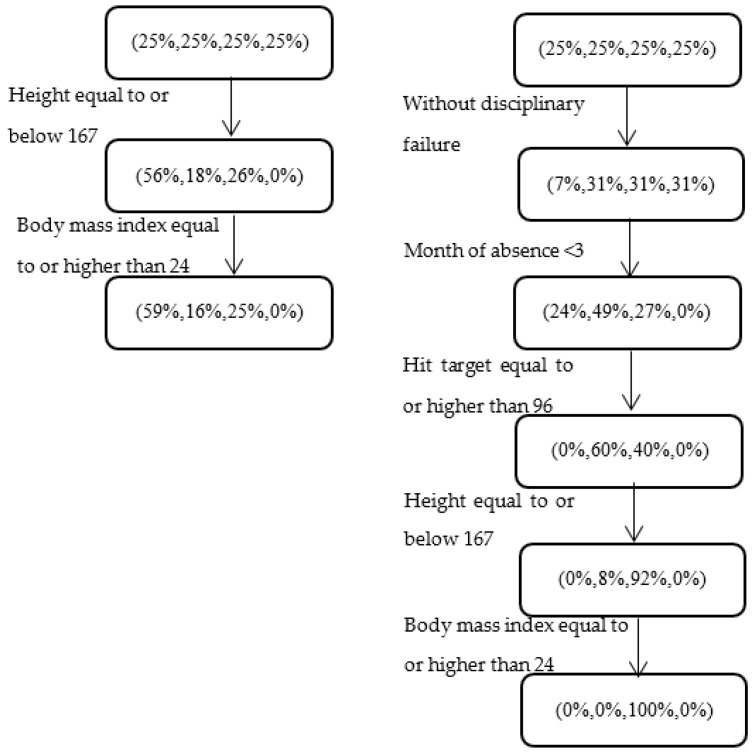
Relationship between body characteristics and absenteeism at work for different subgroups of employees. The LHS and RHS respectively show (i) simple partitions by height and BMI and (ii) a more refined series of patterns revealed by the ordinal CART model.

This example was chosen following previous research studies demonstrating a link between body characteristics (e.g., weight and height) and absenteeism. Figure 3 illustrates a correlation between body characteristics and absenteeism and demonstrates the ability of the ordinal CART to discover refined, multi-feature patterns (RHS), as opposed to partitions based only on body characteristics (LHS). Specifically, it can be seen that for the subgroup of employees who are shorter than or equal to 167cm, with a Body Mass Index (BMI) that is equal to or higher than 24, who do not have a disciplinary failure, and who exhibit relatively high performance, the distribution among the absenteeism classes for absences at the beginning of the year is (0%, 0%, 100%, 0%). In other words, all the instances within this subgroup belonged to the class “days”. 

We note that in both the simple partitioning scheme and the ordinal CART model (LHS and RHS of Figure 3, respectively) the distribution of the level of absenteeism based on height is similar whether or not the information about BMI is included. This is unsurprising given that height is part of the BMI calculation, but it underscores the ability of our model to uncover refined patterns in which multiple features come into play. Specifically, the pattern on the RHS, which corresponds to a subgroup of nine dataset instances, is significantly different (with *p*-value < 0.001) from the pattern obtained for the full set of instances with the same body characteristics (59%, 16%, 25%, 0%). Thus, the ordinal CART enables the identification of a subgroup with significantly higher absenteeism rates. These relatively complex patterns, and their associated interpretations, can be easily lost when the analysis is carried out via noninterpretable models.

With regard to a possible explanation for this finding, we note that BMI values of 25 to 29.9 are considered overweight, and individuals with values above 29.9 are considered obese [8,31]. Furthermore, high BMI values are associated with an increased sickness absence, as shown by previous research [8,9]. It seems that when these employees have a high rate of performance, they are confident and thus more prone to be absent than employees with the same BMI values but a lower rate of performance. A possible intervention policy would consist of an educational program for this subgroup about the negative effects of an elevated BMI and about the importance of healthy food and an active lifestyle. This information could be delivered in a workshop or through individual training sessions.

**Example** **3.** **The relationship between workload and the level of absenteeism.**

**Figure 4:**
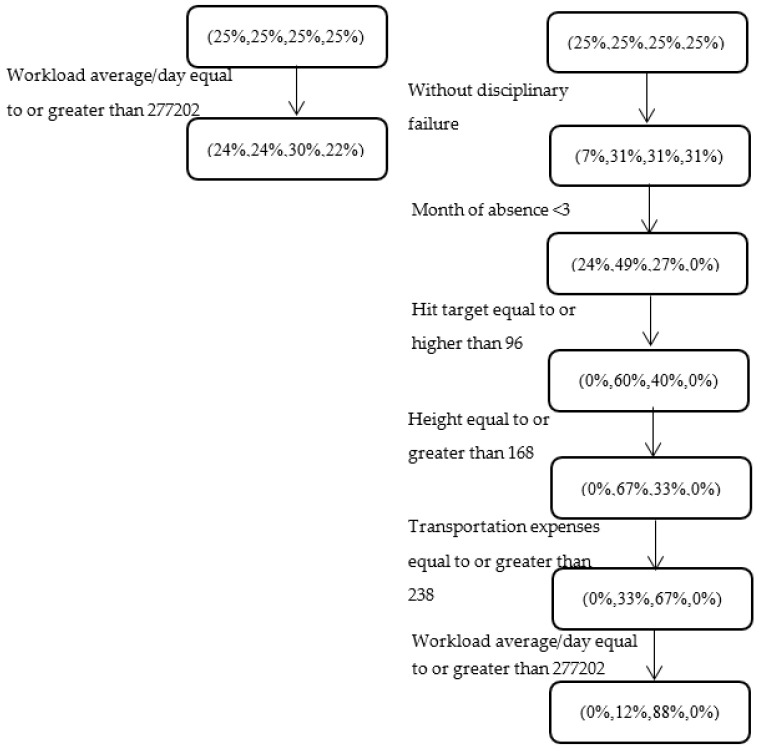
Relationship between workload and absenteeism at work for different subgroups of employees. The LHS and RHS, respectively, show (i) a simple partition by workload and (ii) a more refined series of patterns revealed by the ordinal CART model.

As in previous examples, we focus here on a factor (workload) that has been reported to be associated with absenteeism (see, e.g., in [3]). As can be seen on the RHS of Figure 4, when the period of absence is at the beginning of the year, employees without a disciplinary failure, whose height is equal to or higher than 167cm, with high performance, transportation expenses that are equal to or higher than 238 (the expenses of 58% of instances are lower than this value), and a daily workload that is equal to or higher than 277,202 (the workload of 67% of instances is lower than this value), the distribution among the absenteeism classes is (0%, 12%, 88%, 0%). Thus, 12% of such instances are absent for “hours”, while 88% are absent for “days”. The absenteeism class distribution for this group of 16 dataset instances is significantly different (with *p*-value <0.001) from a classification that is based solely on workload (24%, 24%, 30%, 22%); the latter classification is not very informative for decision makers.

A possible explanation for the discovered pattern may be that overloaded employees who live further from work (as can be deduced from their higher transportation expenses) may gradually burn out and accrue health issues that may lead to absenteeism. The discovered pattern provides additional refinements to previous studies (see, e.g., in [3]) that show that increased workload may be associated with adverse health effects, which, in turn, may lead to increased levels of sickness absence. When examining a pattern that is that same as that on the RHS of Figure 3 but with a difference at the final level (employees with a daily workload that is lower than 277,202), the resulting class distribution of (0%, 67%, 33%, 0%) is significantly different from the (0%, 12%, 88%, 0%) distribution obtained for those with a higher workload. These findings may trigger a discussion about intervention programs that balance workloads or offer flexible work opportunities, such as working from home, which may decrease transportation expenses, save travel time, and potentially increase productivity.

More generally, it is worth noting which features were most influential in the classification of the level of absenteeism. The feature “disciplinary failure” appears as the first feature in all three patterns in the above examples (and in all patterns produced by the ordinal CART), and thus makes the most significant contribution to the classification of absenteeism. The features “month of absence”, “hit target”, and “workload” are the next most influential features, as they appear at depths 2 and 3 of the decision tree (out of 17 depths in total). The third set of features that can be considered influential consists of “height”, “age”, and “BMI”, which appear at depth 4 of the tree. 

**Figure 5:**
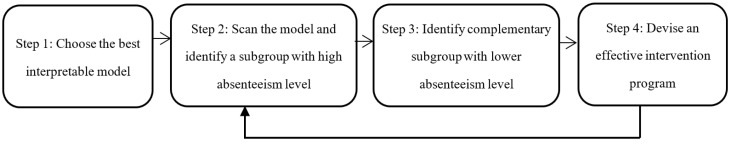
A mechanism for guiding the selection and development of intervention programs for employee subgroups.

We suggest a general mechanism, based on our classification models, for guiding (a) the selection of employee subgroups that are prone to absenteeism and (b) the development of intervention programs to mitigate this behavior (see Figure 5). The first step is to select the best interpretable model, which was found to be the OBE-based CART model with selected class $c^{\max}$. In Step 2, an automatic mechanism identifies an employee subgroup with a high likelihood of absenteeism. Next, in Step 3, the mechanism identifies the complementary subgroup with a lower level of absenteeism. This subgroup has the same pattern as the subgroup that was identified in Step 2, but with a different value for the last feature in the pattern. This complementary subgroup, in conjunction with other human resource management best practices, will be used as an inspiration for assembling the intervention program in Step 4. This is because it is assumed that following the intervention, employees within the high absenteeism subgroup will behave more like those in the complementary subgroup, and thus will be less prone to absenteeism. The examples above (which refer to 3 out of 123 patterns yielded by the model) illustrate how human resource managers can use the suggested approach for reducing absenteeism.

## 4. Conclusions and Discussion

This study develops an objective-based entropy approach for decision tree models. We demonstrate how the approach may be implemented to select the most useful features and identify complex absenteeism patterns. A comparison of the ordinal CART model with other alternatives demonstrates that the proposed model is superior based on a variety of common performance indices. We contend that this superior level of performance, combined with the interpretability capabilities, make this model a very attractive alternative for performing analyses and making predictions in human resource domains that include ordinal data. Specifically, for the present application, the ordinal CART model can be used as a tool to identify subgroups of employees with particular absenteeism patterns. Such discoveries may facilitate understanding of absenteeism phenomena, which, in turn, may lead to selective actions and policies aimed at decreasing absenteeism.

The main contributions of our research are as follows.

(1) Methodology. We introduce a new information measure, known as the objective-based entropy, which extends the weighted entropy proposed in Singer et al. [16] and considers the ordinal nature of the target (in this case, absenteeism). In contrast to standard entropy measures, the objective-based entropy can differentiate between two situations in which the set of absenteeism classes (“non-absent”, “hours”, “days”, “weeks”) has respective probability distributions of $\left(p_{1},p_{2},p_{3},p_{4}\right)$ and $\left(p_{1},p_{2},p_{3},p_{4}\right)$, for example. We demonstrate the use of the new measure and, in particular, highlight its suitability when the objective is to identify a specific class-level (in the present case, those who may be particularly susceptible to absenteeism). Thus, the objective-based entropy measure makes it possible to focus on a specific class, unlike previous approaches that tend to focus on model-level indices (e.g., accuracy).
(2) Modeling. This research highlights the value of interpretable models as decision support tools in applications such as human resource management. Indeed, human users (in our case, human resource managers) prefer interpretable models that enable their reasoning [17,18]. In the current study, understanding the logic of the models may enable human resource managers to take action and devise data-driven policies for decreasing and preventing absenteeism. We provide numerical examples to demonstrate the ability of interpretable models to uncover subgroups of individuals with common characteristics who fall into the same class of the target variable. This approach produces insights that are not discovered through conventional methods, such as hypotheses testing and regression models, as the latter typically focus on high-level correlation between individual features and the target variable (e.g., “absenteeism increases with workload”). Based on this argument, we contend that interpretable models may be superior to their noninterpretable counterparts in terms of organizational benefit, even if their performance is slightly lower. Fortunately, in this research, our interpretable models also achieve higher performance than their noninterpretable counterparts.
(3) Practice. Last, the current study contributes to research on absenteeism by departing from previous research in which the “reason for absence” was used as an explanatory feature. In practice, the reason for absence is not known ahead of the absenteeism event and, moreover, most organizations do not record in their information systems the specific medical situations of their employees. Combined with the use of interpretable models that enable human resource managers to decide on actionable policies, we would argue that our model has greater practical value for analyzing and predicting absenteeism patterns than previous models that did include “reason for absence” as a feature and that were based on non-interpretable models.

As mentioned above, to demonstrate the capabilities of our ordinal interpretable model, we present three example patterns that involve features which are known to be correlated with absenteeism: age, body characteristics, and workload. Using these examples, we show that our model uncovers refined, multi-feature patterns through which human resource managers can pinpoint employee subgroups with distinct absenteeism behavior. These descriptive interpretations may enable human resource managers to take informed actions targeted at specific subgroups rather than general actions aimed at coarse subgroups (e.g., partitioned by age or workload). Specifically, human resource managers can devise a set of intervention programs that is tailored to selected employee subgroups, with the goal of reducing absenteeism.

Two attractive features of the proposed model are (1) the possibility of generalizing it to many other domains—beyond absenteeism and the field of human resources—and (2) the possibility of further tuning the model so as to improve its accuracy as new data are established. Future research directions may include theoretical analyses of the effect of different statistics on the OBE measure (to identify a variety of probability distributions) or adaptations of the OBE to continuous models. Another research direction could be to use the weighting terms proposed in the OBE in conjunction with other measures such as the Fisher score or the Gini impurity measure for ordinal classification purposes. From a practical point of view, it would be interesting to develop additional interpretable objective-based entropy models and evaluate their performance on various human resource-oriented datasets in conjunction with human resource managers.

## Figures and Tables

**Table 1:** Description of the dataset’s features.

Feature Name	Feature Type	Possible Values (for Nominal Variables)
ID	Numerical	
Reason for absence	Categorical	21 categories according to the International Code of Diseases (ICD)
Month of absence	Categorical	1-January 2-February 3-March 4-April 5-May 6-June 7-July 8-August 9-September 10-October 11-November 12-December
Day of the week	Categorical	2-Monday 3-Tuesday 4-Wednesday 5-Thursday 6-Friday
Season	Categorical	1-summer 2-autumn 3-winter 4-spring
Transportation expense	Numerical	
Distance from residence to work (km)	Numerical	
Service time	Numerical	
Age	Numerical	
Workload (average daily)	Numerical	
Hit target	Numerical	
Disciplinary failure	Categorical	1-yes 2-no
Education	Categorical	1-high school 2-graduate 3-postgraduate 4-master/doctor
# of children	Numerical	
Social drinker	Categorical	1-yes 2-no
Social smoker	Categorical	1-yes 2-no
# of pets	Numerical	
Weight	Numerical	
Height	Numerical	
Body mass index	Numerical	
Absenteeism (hours)	Numerical	

**Table 2:** Categorization of absenteeism classes.

Absenteeism Hours (*y*)	Absenteeism Class	c	V(c)	P(c)
0	not absent	$c_{1}$	1	6%
0 < *y* < 8	Hours	$c_{2}$	2	57%
8 ≤ *y* < 40	Days	$c_{3}$	3	34%
*y* ≥ 40	Weeks	$c_{4}$	4	3%

**Table 3:** Distribution of training dataset classes before and after Synthetic Minority Oversampling Technique (SMOTE) implementation.

	Not Absent	Hours	Days	Weeks	Total Instances
Training before SMOTE	6%	57%	34%	3%	592
Training after SMOTE	25%	25%	25%	25%	1360

**Table 4:** Entropy and objective-based entropy (*OBE*) measures with selected statistics $c^{\max}$ and $c^{\mathrm{mode}}$ for two different probability distributions of the absenteeism classes (“not absent”, “hours”, “days”, and “weeks”).

$\left(P(c_{1}),P(c_{2}),P(c_{3}),P(c_{4})\right)$	H(c)	$OBE(c^{\max})$	$OBE(c^{\mathrm{mode}})$
(0.6,0.3,0,0.1)	1.30	0.43	0.25
(0.6,0.1,0,0.3)	1.30	0.38	0.36

**Table 5:** Average performance measures of different learning models for the absenteeism at work dataset.

	Performance Measures						
	F-score	Precision	Recall	Accuracy	AUC	MSE	$\tau_{b}$
*Non-ordinal classifiers*							
Extreme Gradient Boosting (XGBoost)	0.69	0.72	0.68	0.68	0.73	0.32	0.52
Multi-Layer Perceptron (MLP)	0.42	0.33	0.57	0.57	0.50	0.49	0.40
K-Nearest Neighbor	0.56	0.56	0.56	0.56	0.60	0.58	0.35
Naïve Bayes	0.41	0.54	0.34	0.34	0.56	1.46	0.02
Random Forest (RF)	0.67	0.68	0.67	0.67	0.70	0.35	0.51
CART	0.66	0.66	0.66	0.66	0.69	0.36	0.41
*Ordinal classifiers*							
Ordinal CART $OBE(c^{\mathrm{mode}})$	0.69	0.70	0.69	0.69	0.72	**0.31**	0.53
Ordinal CART $OBE(c^{\max})$	**0.73**	**0.74**	**0.72**	**0.72**	**0.76**	0.34	**0.58**
